# Metabolomics Analysis of the Prefrontal Cortex in a Rat Chronic Unpredictable Mild Stress Model of Depression

**DOI:** 10.3389/fpsyt.2022.815211

**Published:** 2022-03-15

**Authors:** Lihua Duan, Rong Fan, Teng Li, Zhaoyu Yang, En Hu, Zhe Yu, Jing Tian, Weikang Luo, Chunhu Zhang

**Affiliations:** ^1^Department of Integrated Traditional Chinese and Western Medicine, Institute of Integrative Medicine, Xiangya Hospital, Central South University, Changsha, China; ^2^National Clinical Research Center for Geriatric Disorders, Xiangya Hospital, Central South University, Changsha, China

**Keywords:** metabolomics, depression, LC-MS/MS, chronic unpredictable mild stress, prefrontal cortex, metabolite-protein interaction

## Abstract

**Background:**

Depressive disorder is the leading cause of disability and suicidality worldwide. Metabolites are considered indicators and regulators of depression. However, the pathophysiology of the prefrontal cortex (PFC) in depression remains unclear.

**Methods:**

A chronic unpredictable mild stress (CUMS) model and a maturation rodent model of depression was used to investigate metabolic changes in the PFC. Eighteen male Sprague-Dawley rats were randomly divided into CUMS and control groups. The sucrose preference test (SPT) and forced swimming test (FST) were employed to evaluate and record depression-associated behaviors and changes in body weight (BW). High-performance liquid chromatography–tandem mass spectrometry was applied to test metabolites in rat PFC. Furthermore, principal component analysis and orthogonal partial least-squares discriminant analysis were employed to identify differentially abundant metabolites. Metabolic pathways were analyzed using MetaboAnalyst. Finally, a metabolite-protein interaction network was established to illustrate the function of differential metabolites.

**Results:**

SPT and FST results confirmed successful establishment of the CUMS-induced depression-like behavior model in rats. Five metabolites, including 1-methylnicotinamide, 3-methylhistidine, acetylcholine, glycerophospho-N-palmitoyl ethanolamine, α-D-mannose 1-phosphate, were identified as potential biomarkers of depression. Four pathways changed in the CUMS group. Metabolite-protein interaction analysis revealed that 10 pathways play roles in the metabolism of depression.

**Conclusion:**

Five potential biomarkers were identified in the PFC and metabolite-protein interactions associated with metabolic pathophysiological processes were explored using the CUMS model. The results of this study will assist physicians and scientists in discovering potential diagnostic markers and novel therapeutic targets for depression.

## Introduction

Depression is a severe psychiatric disease characterized by a significant and lasting depressed mood (1). Depression may lead to various critical function disorders, including insomnia, addiction, neurodegenerative diseases, and other complications, which can severely affect a patient's physical and mental health. Moreover, depression is one of the main causes of disability and even death worldwide (2), imparting extraordinary economic burdens upon society (3). Despite considerable evidence regarding the pathophysiology of depression, the fundamental molecular processes that mediate its onset are still unknown. Therefore, it is necessary to explore the pathophysiologic mechanisms of depression to identify potential therapeutic targets.

Due to the difficulty of obtaining human cortex specimens, comprehensive metabolomics analyses of the prefrontal cortex (PFC) are lacking, even though the PFC plays a vital role in neural pathways related to emotion, cognition, and learned responses (4). A large number of functional imaging, lesion, and brain stimulation studies have indicated that the PFC is essential for depression (5). Metabolic dysregulation in the PFC in depressed patients or model animals has been reported (6, 7). For example, in adult humans, BDNF (brain-derived neurotrophic factor) expression is decreased in the hippocampus and PFC upon exposure to stress and depression, and antidepressant treatment up-regulates BDNF expression (8). In mouse models of depression, chronic unpredictable moderate stress–induced behavioral alterations are associated with dopaminergic hyperfunction and serotonergic hypofunction (9). Numerous studies have reported that central levels of monoamine neurotransmitters, including serotonin, dopamine, and norepinephrine, are down-regulated, supporting the classical monoamine hypothesis of depression (10). Moreover, increased glutamate levels can cause neurotoxicity and nerve damage in patients with depression (11). A decline in serotonin levels is the main feature of depression (12). Some previous studies found that depressive rats showed amino acid–related metabolic dysfunction only in the PFC (13). Patients with depression disorder are taking the above recognized and classic antidepressants, but the treatment effect is not good (14). Hence, a large part of the reason may be that the pathogenesis of depression was vague. It is particularly important to characterize the cortical metabolism of depression.

Metabolomics is an emerging area of study in systems biology research that focuses on the discovery of biomarkers and the elucidation of biological processes controlling metabolites (15). An important benefit of metabolomics over proteomics methodologies is the direct biological closeness to the system's phenotype and the capacity to monitor rapidly occurring metabolome alterations (16). Metabolomics approaches are increasingly utilized to diagnose illnesses and identify biomarkers (17). For example, liquid chromatography–mass spectrometry (LC-MS) has been used to profile serum metabolites in depression and characterize the association between differential metabolites and neurocognitive dysfunction (18, 19).

In this study, we focused on the metabolic changes in the PFC in model rats subjected to chronic unpredictable mild stress (CUMS). To identify significantly altered metabolic pathways and explain the pathogenesis of depression, we further established the associated metabolite-protein network via database analyses to avoid biases inherent in traditional methods. Using this approach, significant metabolite functions in the PFC were excavated from the data. Protein target prediction based on metabolite structure is worthy for exploring novel targets and functions.

## Materials and Methods

### Animals and Reagents

All experiments and procedures followed a protocol approved by the local Animal Ethics Committee of the Institution of Research Animal Care of Central South University and observed the principles of laboratory animal care (approval ID: 2020SYDW0892). Eighteen healthy male Sprague-Dawley rats were acquired from the Experimental Animals Center of Central South University, Changsha, Hunan. Each rat weighed between 180–220 g at the beginning of the experiment. The animals were habituated for no <7 days before being subjected to CUMS. The feeding conditions during the adaptation period are as follows: three rats were housed per cage under specific pathogen-free conditions and 12/12 h light-dark cycle (0800–2,000), background control range noise (40 ± 10 dB), and temperature (20 ± 3°C), with food and purified water accessible ad libitum. After 7 days of habituation, the rats were randomly assigned to two groups by ballot: control group and CUMS group (*n* = 9 per group). The normal control rats were housed three per cage, whereas rats in the CUMS group were individually caged.

Methanol, ammonium hydroxide, and ammonium acetate were obtained from Sigma-Aldrich (St. Louis, MO, USA). Water and acetonitrile (ACN) were purchased from J.T. Baker (PA, USA). Other reagents were of analytical grade.

### Establishment of CUMS Rat Model

CUMS was carried out as reported for other studies (20, 21). After the 7-day habituation period, the rats in the control group were given food and water normally, except that water was withheld for 24 h before the sucrose preference test (SPT). CUMS-induced depression rats were isolated in individual cages and randomly subjected to the following conditions for 4 weeks: food deprivation (24 h), water deprivation (24 h), level shaking for 5 min (1 time/s), 45° cage tilting (8 h), restraint in an empty water bottle (Yi-bao, China) (4 h), wet bedding for 24 h (200 mL of water per individual cage to make the bedding wet), noise (20 min), tail clamp for 2 min, swimming in cold (4°C) water (5 min), swimming in hot (40°C) water (5 min), and day-night switch (reversal of 12/12 h light/dark cycle). To ensure the procedure was unpredictable, the above stimulations were applied to CUMS rats randomly and discontinuously (1 type per day) over the course of 28 days. After the experiment, rats were sacrificed under intraperitoneal injection of 3% pentobarbital sodium (60 mg/kg) anesthesia on day 28. The PFC was isolated on an iced plate and stored at −80°C.

### Behavioral Tests

On days 1, 14, and 28 after the operation, CUMS was evaluated using the SPT and the forced swim test (FST) as previously reported with minor modifications (22). In addition, rat body weight (BW) was measured. Animals were arranged in the testing room and adapted for 0.5 h before the test. All tests were performed in a dim and quiet room. After testing, the rats were returned to the holding rooms.

### SPT and BW Changes in Model Rats

The SPT was performed with reference to previous literature (23). In brief, rats were trained to adapt to sucrose solution (1%, w/v) before testing. Two bottles of 1% sugar water were available for each cage, one of which was exchanged with distilled water 24 h later. After adaption, the 24-h supply of water and food were removed from each cage. Rats were then kept in separate cages with free access to the two bottles, one containing 200 mL of sucrose solution (1%, w/v) and the another containing 200 mL of pure water. The level of the bottles was balanced, and the two bottles were exchanged after 2 h to avoid side preference. The weight of the bottles was measured before and after the 4-h test. After the test was completed, the sucrose preference was calculated using the following formula: sucrose preference (%) = sucrose consumption/(total consumption [sucrose + water]) ×100% (24). All rats were weighed at the three-time points mentioned above and the rate of weight change was calculated.

### FST

The FST was carried out with reference to a previous method (25). The FST remains one of the most commonly used tools for screening CUMS-induced depression due to its good predictive performance, rapidity, and cost-effectiveness. Swimming initiated by placing the rats in cylinders (46 cm height ×45 cm diameter) containing 25°C water at 41 cm in depth so that the rats could not support themselves by touching the bottom with their feet. The rats were exposed to a pre-test for 15 min, and the next day they were subjected to the FST. The experiment was conducted for 5 min, after which immobility time was recorded. Floating in the water without struggling but exhibiting movements necessary to maintain the head above the water was regarded as immobility.

### Sample Preparation

PFC tissue (45 mg) was homogenized with 300 mL of pure H_2_O. To measure the total protein concentration of each individual homogenate, a BCA protein assay was carried out. Metabolites were extracted from the homogenates using a 1:1 (v/v) mixture of methanol and ACN. After vortexing for 30 s and sonicating for 10 min, the samples were incubated for 1 h at −20°C and centrifuged at 20,000 rpm at 4°C. The supernatants were collected and dried in a vacuum concentrator. Finally, the dry extracts were reconstituted in 40 μL/mg protein using ACN:H_2_O (1:1, v/v), vortexed for 30 s, and sonicated for 10 min prior to LC-MS analysis. Pooled quality control (QC) samples were prepared by combining 10 mL equal volumes from each sample (one QC sample per four samples). The extraction of QC samples was the same as that for test samples.

### LC-MS/MS Analysis

Samples were separately tested using a 1260 Infinity liquid chromatograph (Agilent J & W Scientific, Folsom, CA, USA) coupled with a Q-Exactive MS/MS (Thermo, MA, USA) under both positive and negative ion modes. Chromatographic separations were performed on an amide column at 25°C using a combination of water and 25 mM ammonium acetate and 25 mM ammonium hydroxide as mobile phase A and ACN as mobile phase B. A 4-μL sample was injected at a rate of 0.4 mL/min. Relevant tuning parameters for the probe were set as follows: aux gas heater temperature, 400°C; sheath gas, 40 psi; auxiliary gas, 13 psi; spray voltage, 3.5 kV for positive mode and negative mode. The capillary temperature was set at 350°C. The data-dependent acquisition method was established as listed: total scan range: 60–900 *m/z*; resolution: 140,000; maximum injection time: 100 ms; automatic gain control: 3 ×10^6^ ions.

### Data Processing

Raw LC-MS data were first processed using Compound Discover 2.0 (Thermo). The Compound Discover software identifies metabolic features with reproducible differences across multiple sample groups. BioCyc, Human Metabolome Database, Kyoto Encyclopedia of Genes and Genomes (KEGG), and McCloud databases were searched to assist with metabolite identification. Only metabolites that were matched with the standard ddMS2 spectrum were subjected to further analysis. The usable data matrix, including *m/z*, retention time, and intensity, was imported into SIMCA-P software (version 14.1, Umetrics, Umea, Sweden) and subjected to multivariate statistical analysis. Unsupervised principal component analysis (PCA), supervised partial least squares discriminant analysis (PLS-DA), and orthogonal PLS-DA (OPLS-DA) were performed. Variables important in the projection (VIP) were calculated from the PLS-DA model. Metabolites with a VIP >1 and *p*-value from Student's *t*-test of <0.05 were viewed as differently expressed metabolites. The results were then visualized using R (version 3.5.2).

Next, MetaboAnalyst version 5.0 (https://www.metaboanalyst.ca/) was used for pathway enrichment analysis. Names of differentially expressed metabolites were imputed, and “*Rattus norvegicus* (rat)” was chosen as the pathway library. Differentially expressed metabolite–associated proteins, including enzymes, transporters, receptors, and predicted targets in rat species were identified using SEA (http://sea.bkslab.org/) based on maximum Tanimoto coefficients >0.57, STITCH (http://stitch.embl.de/), and Swiss Target prediction (http://www.swisstargetprediction.ch/) based on probability greater than zero. Furthermore, we searched for depression targets via the OMIM (https://omim.org/), Genecards (https://www.genecards.org/), and Disgenet databases (https://ngdc.cncb.ac.cn/databasecommons/). The intersections of disease targets and proteins were predicted based on metabolites (26). Conserved gene-based proteins were selected from among the above proteins using BLAST-NCBI2 for each protein with >80% mRNA identification (27). Finally, the results of metabolite-protein network analysis, KEGG pathway analysis, and further biological analyses were visualized using Cytoscape 3.7.2.

### Western Blot

PFC tissues were washed by ice-cold PBS and lysed in RIPA buffer, followed by homogenizing mechanically. The homogenates were centrifuged at 12,000 rpm for 15 min at 4°C. After electrophoresis on SDS-PAGE gels, proteins were transferred to membranes and blocked with 5% non-fat milk. Then, the blots were incubated with poly (ADP-ribose) polymerase 1 (Parp1) antibody (wx785984, 1:1000; ABclonal, China), glutamate receptor 2 precursor (Gria2) antibody (r22839, 1:1000; zen-bio, China) and mouse-anti rat β-actin antibody (66009-1-Ig, 1:5000; Proteintech, USA) at room temperature. After washing with PBST three times, the blots were incubated with HRP goat-anti-rabbit IgG (SA00001-2, 1:6000; Proteintech, USA) for 90 min. The color reaction was performed by ECL reagents. The band density was visualized after exposure to x-ray film and analyzed using the quantity one software (Bio-Rad, USA).

### Statistical Analysis

SPSS 26.0 (International Business Machines Corp., Armonk, NY, USA) was used analyze the results. To assess normality and equality of variance, we used the Kolmogorov-Smirnov test and Levene's test. Determination of whether to use the Student's *t*-test was based on whether the data exhibited normal distribution; for unequal variance, the data were analyzed using the Mann-Whitney *U*-test, and Fisher's exact test was used to compare the statistical significance of differences between groups. The level of statistical significance was set at *p* < 0.05 (two-sided) for all tests. The results of behavioral tests were depicted using GraphPad software (version 11.0, San Diego, CA, USA).

## Results

### CUMS Model Rats Exhibited Depression-Like Phenotypes

**Figure 1:**
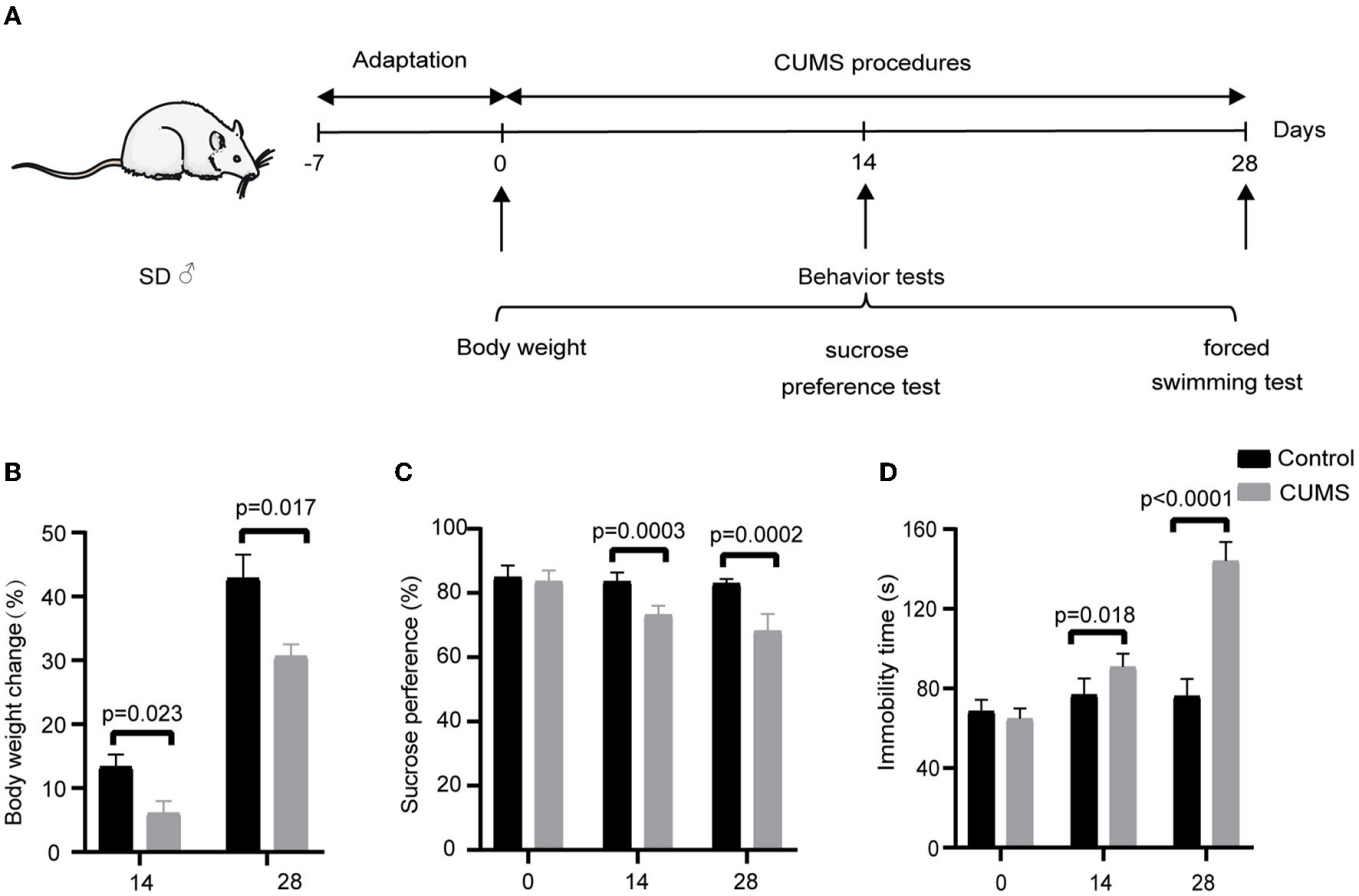
**(A)** Schedule of CUMS exposure; depression-like behavior was examined at days 0, 14, 28 (Mean ± SD, *n* = 5) by **(B)** body weight change measurement, **(C)** sucrose preference test, and **(D)** forced swimming test. CUMS group vs. control group.

The schedule of CUMS is shown in Figure 1A. There were no significant differences in baseline BW before application of CUMS (Supplementary Figure 1). After 14 days of exposure, a significant difference in BW emerged between the CUMS group and control group (*p* < 0.05). At day 28 of CUMS, the BW of rats in the CUMS group exhibited less of an increase compared with the control group (*p* < 0.05), as shown in Figure 1B.

Before CUMS exposure, the baseline sucrose preference index between the control group and CUMS group exhibited no significant difference. On day 14 of CUMS exposure, the sucrose preference index of rats in the CUMS group was significantly lower than that of the control group (*p* < 0.01). On day 28 of CUMS exposure, the sucrose preference results were the same as on day 14 (*p* < 0.01), as shown in Figure 1C.

As with the above tests, there was no significant difference between the two groups before CUMS modeling in the mean immobility time as measured by FST. However, 14 days of exposure to CUMS led to a longer mean immobility time in the FST compared with the control group (*p* < 0.05). At day 28 of CUMS exposure, the immobility time differed significantly between the two groups, as shown in Figure 1D (*p* < 0.01), indicating that the model of depression was successfully established.

### Monitoring the Stability of Sample Preparation

The metabolic features in the PFC were detected. A total of 112 compounds were identified by comparison with the database ddMS2. We used PCA to assess the deviation variance of all QC samples. A plot of 95 percent confidence intervals showed that all of the QC samples fell within the 2SD region (Supplementary Figure 2). This result showed that our experimental settings were adequate for processing metabolomics data from experimental samples.

### Identification of Metabolite Biomarkers Discriminating the CUMS and Control Groups

**Figure 2:**
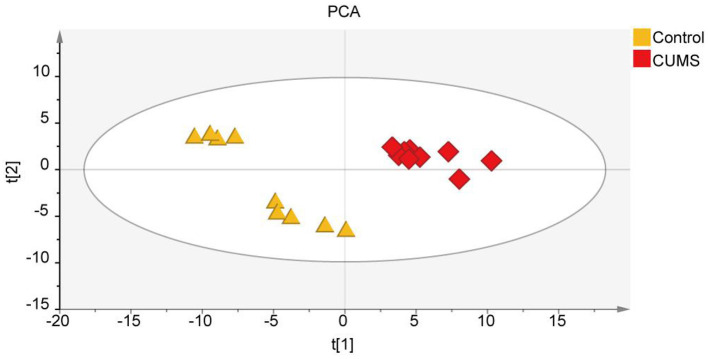
Non-targeted metabolomic analysis of all samples from rats in the principal component analysis (PCA) including the CUMS group and control group; R2X = 72%, Q2 = 54%, *n* = 9.

**Figure 3:**
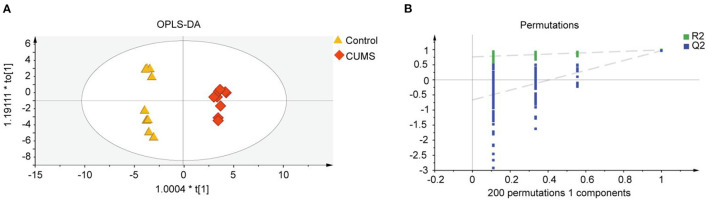
Metabolomic fingerprint analysis of the PFC in orthogonal partial least-squares discriminant analysis (OPLS-DA). **(A)** OPLS-DA score scatter plot, with orange triangles and red diamonds representing the Control group and CUMS group, respectively; R2X [1] = 70%, R2Y [2] = 99.2%, Q2 = 96.8%. **(B)** Validation plot obtained from 200 permutation tests for the OPLS-DA model. R2 = 76.2% and Q2 = −66.6%, *n* = 9.

To explore the differences in metabolite expression between the CUMS and control groups, PCA, PLS-DA, and OPLS-DA models were established. A score plot of PCA (Figure 2) was generated, which revealed that each group exhibited a tendency to gather (R2X = 72%, Q2 = 54%). To maximize the ability to distinguish the CUMS and control groups, an OPLS-DA model was established, which revealed that the CUMS group could be clearly separated from the control group. The R2X, R2Y, and Q2 values in the OPLS-DA model were 70, 99.2, and 96.8%, respectively, and the 200 random permutation test R2 and Q2 values were 76.2 and −66.6%, respectively (Figure 3). The OPLS-DA model showed that the Q2 value was negative, meaning that the model exhibited good explanatory ability regarding sample classification information and cross-validated predictive capability.

**Figure 4:**
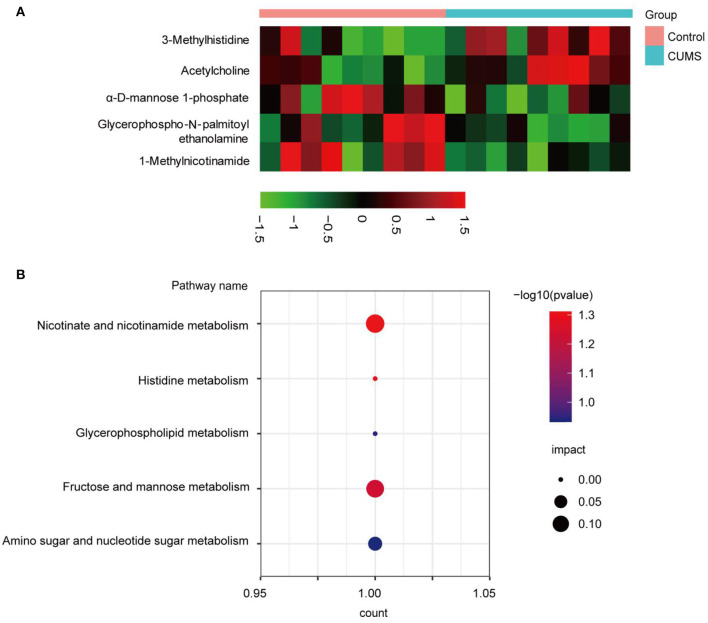
Bioinformatic analysis of significantly differentially expressed metabolites. **(A)** Red indicates significantly up-regulated metabolites, whereas black indicates significantly down-regulated metabolites in the PFC. **(B)** Bubble chart of the metabolic pathway analysis. The *p*-value and impact factor are represented by dot color and dot size, respectively. The count represents total number of hits in the metabolic pathway.

To screen differential metabolites between the CUMS and control groups, five significant metabolites were determined based upon VIP >1.0 and *p* < 0.05, including 1-methylnicotinamide (MNA), 3-methylhistidine (3-MH), acetylcholine (Ach), glycerophospho-N-palmitoyl ethanolamine (GP-NPEA), and α-D-mannose 1-phosphate (Man-1-P). Detailed information regarding these metabolites is shown in Table 1. The ddMS2 spectra of the differential metabolites are displayed in Supplementary Figure 3. To visualize metabolite expression between the two groups, a heatmap displaying up- (red) and down-regulated (black) metabolites was generated (Figure 4A). Levels of Ach and 3-MH increased, whereas levels of MNA, Man-1-P, and GP-NPEA decreased after CUMS exposure.

**Table 1:** Differential metabolites in CUMS rats.

**Metabolite**	***m/z***	**RT (min)**	**Formula**	**CUMS vs. Control**		
				**VIP**	***P*-value**	**FC**
3-Methylhistidine	169.09	19.34	C_7_ H_11_ N_3_ O_2_	1.1	1.89E-02	1.41
1-Methylnicotinamide	136.06	7.97	C_7_ H_8_ N_2_ O	1.1	1.61E-02	0.88
Glycerophospho-N-palmitoyl ethanolamine	453.29	2.87	C_21_ H_44_ N O_7_ P	1.0	2.79E-02	0.68
Acetylcholine	145.11	3.31	C_7_ H_15_ N O_2_	1.2	1.08E-02	1.81
α-D-Mannose 1-phosphate	260.03	21.00	C_6_ H_13_ O_9_ P	1.2	1.18E-02	0.84

### Analysis of Metabolic Pathways and Integrated Metabolite-Protein Network

To explore the metabolic pathways related to depression, differentially expressed metabolites were imported into MetaboAnalyst 5.0 for analysis of metabolic pathway enrichment. Five pathways (Figure 4B) were regulated in the PFC. Relevant pathways were identified based on an impact value threshold of >0.1 and *p* < 0.05. Nicotinate and nicotinamide metabolism were evident in the PFC (Supplementary Table 1).

**Figure 5:**
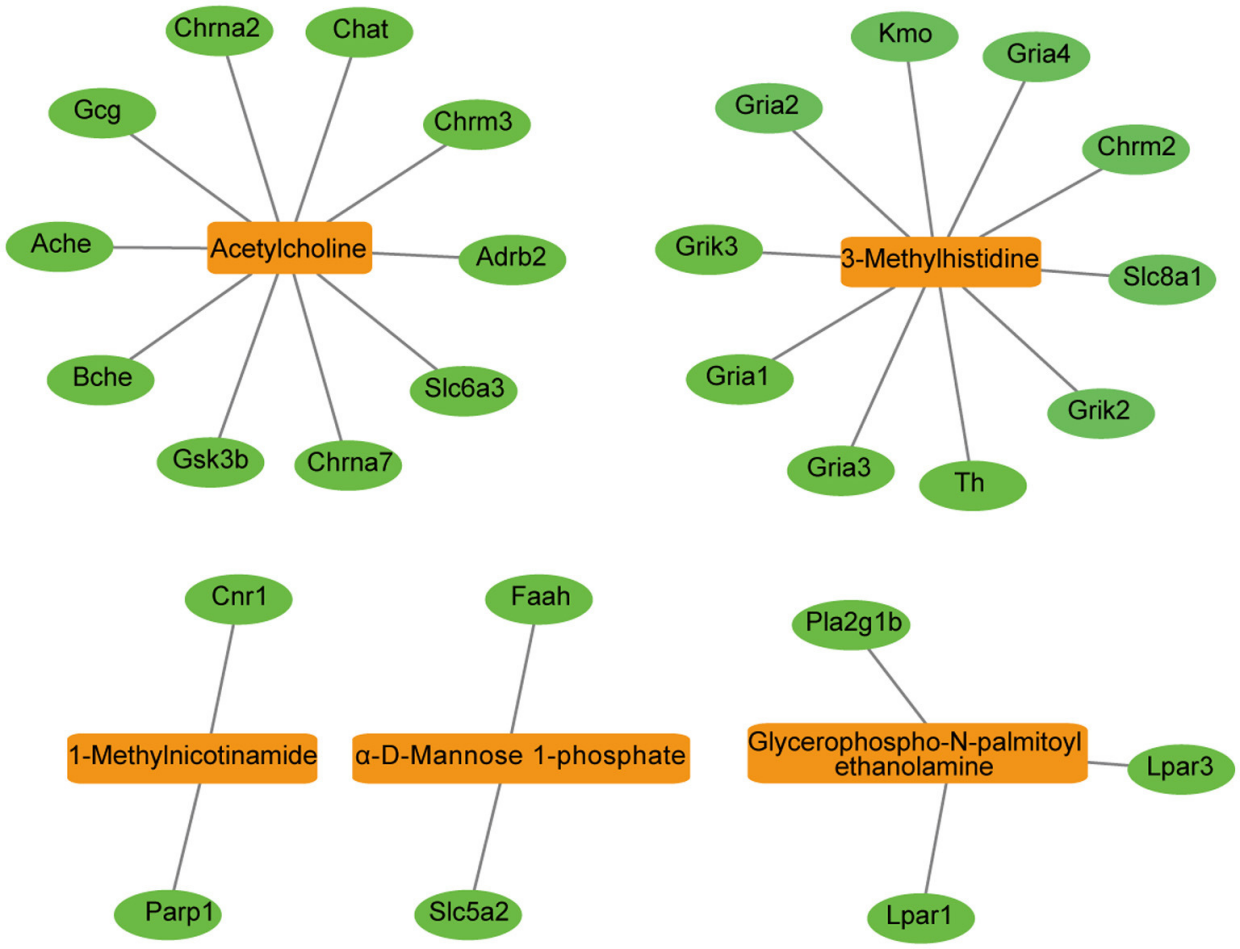
Metabolite-protein network of the PFC visualized using the Cytoscape tool. The network was established with the nodes of metabolite compounds (orange rectangles) and protein enzymes (green ovals).

**Figure 6:**
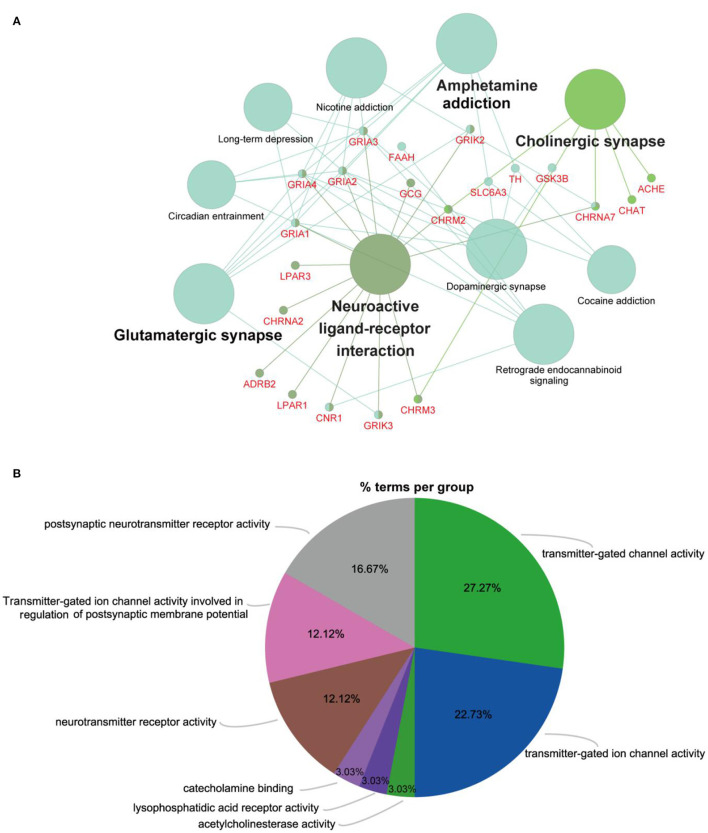
Predicted metabolites and related proteins identified from the database and their associated analysis in the PFC. **(A)** KEGG pathway enrichment analysis was carried out using ClueGO. All pathways shown with a *p* < 0.05. **(B)** Pie chart providing a visualization of the results of GO enrichment analysis of proteins. (For interpretation of the references to color in this figure, the reader is referred to the web version of this article.)

To further evaluate the roles of the filtered metabolites in the pathophysiology of depression, depression-related targets were matched with metabolite-related targets. Comparing BLAST-NCBI2 for each protein, a target of mRNA identification of >80% was selected. Finally, 27 proteins were found to interact with five altered metabolites in the PFC. All of these proteins were transformed into their UniProt symbols using the UniProt database, and a metabolite-protein network was constructed (Figure 5). Furthermore, to elucidate the functions of the potential targets in detail, we performed GO and KEGG pathway enrichment analyses using ClueGO (Figures 6A,B). Ten KEGG pathways were enriched in the PFC. The pathways (Figure 6A) neuroactive ligand-receptor interaction, cholinergic synapse, glutamatergic synapse, and dopaminergic synapse were significantly affected (*p* < 0.05). The top terms in the GO analysis were transmitter-gated channel activity, transmitter-gated ion channel activity, acetylcholinesterase activity, lysophosphatidic acid receptor activity, catecholamine binding, neurotransmitter receptor activity, transmitter-gated ion channel activity involved in the regulation of postsynaptic membrane potential, and postsynaptic neurotransmitter receptor activity (Figure 6B). Detailed information were shown in Supplementary Table 2.

### Verification of the Metabolites-Related Proteins

Combining the metabolites-proteins network analysis with Search literature, we selected that Parp1 and Gria2 may act as a significant effect during the process of CUMS. We examined the expression of Parp1 and Gria2 in the cortex by western blot (Supplementary Figure 4). Compared with the control group, the level of Parp1 and Gria2 down-regulation. These results are consistent with the examination of metabolomics.

## Discussion

The CUMS depression model is recognized as reliable and practical, and thus, it is widely used to study the mechanism of depression (28). In this study, the sucrose preference index in the CUMS group declined significantly, suggesting that the response of rats to rewards weakened and that the depression model was established successfully. The immobility time in the FST reflects the degree of despair in the rats (29). In keeping with previous studies, CUMS significantly prolonged the immobility time in the FST. Hence, analyses of SPT and FST results showed that the rats in the CUMS group exhibited obvious depressive-like behaviors, indicating successful establishment of the CUMS model. Similarly, depression-like symptoms in CUMS rats were accompanied by slow weight gain, which suggests that CUMS probably caused gastrointestinal dysfunction, in turn contributing to a loss of appetite and slow weight gain in the rats. Moreover, study demonstrated that gut microbiota can physiologically induce depression-like behavior in mice (30). Fecal microbiota transplantation of germ-free mice with “depression microbiota” derived from major depressive disorder (MDD) patients resulted in depression-like behaviors compared with colonization with “healthy microbiota” derived from healthy control individuals (31). To explore the metabolic signatures of the PFC, we identified five significantly varying metabolites associated with the metabolic pathways of nicotinate and nicotinamide metabolism. Metabolite-protein interaction analysis revealed that 10 pathways in the PFC are significantly associated with depression (Figure 6A), especially the neuroactive ligand-receptor interaction, cholinergic synapse, glutamatergic synapse, and dopaminergic synapse pathways. These metabolites and metabolite-protein networks provide the basis for a more in-depth understanding of the metabolomic characteristics of depression and potential therapeutic targets.

Note that the cortex is the key functional location in depression (32). Long-term functional defects of the cortex make depression more likely to occur and disease-dependent metabolomic changes in the PFC during the depression have been documented (33). Hence, we identified candidate metabolites and relevant pathways to further elucidate the molecular mechanism of depression. Using LC-MS, we enhanced our understanding of the metabolic pattern changes in the PFC resulting from CUMS and predicted the functions of differential metabolites by establishing a metabolite-protein network.

Nicotinate and nicotinamide metabolism was found to be strongly associated with depression in a metabolomics study of urine and plasma (34). MNA, a component of nicotinate and nicotinamide metabolism, is the main metabolite of nicotinamide (NAM) (35, 36). The pyridine compound NA, which is methylated by nicotinamide N-methyltransferase, an enzyme found predominantly in the liver, contributes to the formation of MNA. Previous studies identified MNA as a neuro-protective metabolite that regulates both thrombotic and inflammatory processes in the cardiovascular system (37, 38). Additionally, injection of MNA not only reduces CUMS-induced depressive-like behaviors but also moderates the effects of CUMS on the hippocampus BDNF signaling cascade in mice. Both BDNF dysfunction and neuroinflammation have been found in depression (39). Furthermore, K252a, a BDNF pathway blocker, effectively reduces MNA-associated antidepressant-like effects in mice (40). Mu et al. found that MNA improves lipopolysaccharide–induced cognitive deficits and memory impairment via inhibition of neuroinflammatory responses and neuronal apoptosis (41). Increased MNA intake from the outside can relieve depression (40). There was evidence reported that NAM has an ability to augment Parp1 activation. NAM is a transient inhibitor of class III histone deacetylase silent mating type information regulation 2 homologs (SIRTs) and SIRT1 is an inhibitor of Parp1. Parp1 has a role in improving validation, its associated anti-inflammatory effects mediated through the induction of BCL6, with the concomitant down regulation of Cyclooxygenase-2 (42). Here, we found that the level of MNA and Parp1 were significantly decreased within the PFC of CUMS rats, consistent with previous findings. Therefore, depression-like behavior may be related to the down-regulation of MNA. It is hypothesized that MNA cannot effectively resist the inflammatory factors produced in depression disorder or limit the effect of BDNF regulation in the PFC.

MH is an amino acid formed by post-translational modification of myofibrillar proteins, which is achieved by the methylation of histidine molecules (43). Evidence suggests that 3-MH could be used as a diagnostic biomarker in human serum for phenotyping depression (44). In olfactory bulbectomy (OB) mice, a valid murine model of agitated depression, OB mice exhibited increased serum concentrations of 3-MH compared with their sham-treated counterparts (43). When OB mice received imaging methods treatment, 3-MH levels decreased. Further, heath et al. demonstrated that 3-MH is engaged in the glutamate and gamma-aminobutyric acid (GABA) metabolic signaling pathways (43). Data indicate lower GABA levels in the vmPFC in MDD, consistent with lower GABA in active depression but not MDD in remission (45). In addition, the alpha-amino-3-hydroxy-5-methyl-4-isoxazolepropionic acid receptor (AMPAR) subunit Gria2 related with MH protein levels were increased upon ketamine treatment (46). In this study, the level of 3-MH was up-regulated and the lower expression of Gria2, compared with the control group, was consistent with other reports (43, 46). Therefore, the occurrence of this phenomenon may be connected with a perturbation in histidine metabolism in CUMS rats. Our research adds to the evidence for 3-MH as a biomarker of depression.

ACh is a potent regulator of neuronal activity throughout the peripheral and central nervous systems and it is essential for a variety of complex behaviors, such as attention and learning tasks (47–49). Some studies have found that levels of ACh are significantly decreased in depressed mice (50). Up-regulated ACh signaling can contribute to symptoms linked with anxiety and depression (51). Peripheral intake of the acetylcholinesterase antagonist physostigmine causes symptoms of anxiety and depression in humans by decreasing the breakdown of ACh and increasing levels of the neurotransmitter in the brain (52). Stress causes ACh release in the hippocampus and PFC (53), but not in the amygdala (54). Possibly because the medial septal neurons that innervate the amygdala have a high basal firing rate (55), and stress cannot elevate ACh levels higher. Likewise, due to tonic firing of intrinsic cholinergic interneurons, basal ACh tone in the striatum is high and behaviorally relevant stimuli cause a stop in their firing, resulting in cue-dependent learning (53). These findings show that healthy behavior relies on properly balanced cholinergic signaling across brain regions. In our research, the level of ACh was increased, indicating excessive activation of cholinergic signals acts in the PFC and probably leading to depression.

GP-NPEA is the metabolic precursor of palmitoyl ethanolamide (PEA), an endogenous cannabinoid found in the liver, brain, and other mammalian tissues that has potent anti-inflammatory activity. PEA has a low affinity for central cannabinoid (CB1) receptors for CB2 (56, 57). CB1 agonists reduce excitotoxicity, whereas CB2 agonists limit the toxicity of reactive microglia and antioxidant cannabinoids attenuate oxidative damage. Accordingly, the effect of PEA is mediated via a different receptor (58). The decrease in GP-NPEA in the cortex of CUMS rats may be related to a disorder in the endocannabinoid system arising after the onset of depression.

Man-1-P is a normal metabolic intermediate in fructose and mannose metabolism and a fructose-producing substrate. According to previous research, fructose has memory-enhancing qualities (59) and can function as a neuroprotectant under specific conditions (60). These findings suggest that fructose has direct actions in the central nervous system. Liu found that Man-1-P levels decreased in social defeat stressed rats with metabolite changes in the prefrontal cortex (61). Our results are in conformity with previous studies, in that they indicate Man-1-P may participate in depression pathophysiology through fructose and mannose metabolism, amino sugars, and nucleotide sugar metabolism. Here, we provide evidence of perturbations in Man-1-P metabolism in the PFC metabolites in rats with depression.

The enriched metabolite-protein pathways in this study are directly related to depression. With dopaminergic synapses, the cause of dopaminergic system deficits is likely dysregulation of the system's afferent regulatory circuits following activation of the particular pathway and inhibition of the BLA-VP pathway, resulting in depression (62). Other studies have linked the muscarinic cholinergic receptor system to the pathophysiology of depression, with physiological evidence indicating that this system is overactive or hyperresponsive in depression (63). In our research, ACh levels increased and further enriched cholinergic synapse and acetylcholinesterase activity simultaneously with metabolite-protein functions, consistent with the above reports. Janowsky reported that boosting cholinergic activity using the anticholinesterase inhibitor physostigmine was capable of both generating and worsening depressive symptoms in patients with MDD (64). Consequently, the metabolite-protein network provides a basis for understanding the molecular mechanisms of depression in detail. These results provide a global view of the interactions between metabolites and related proteins, which may help guide the treatment of depression.

## Conclusion

In this study, we revealed significant perturbations in the levels of 3-MH, MNA, GP-NPEA, ACh, and Man-1-P in the PFC of CUMS rats. Furthermore, Parp1 and Gria2 were verified down-regulation in the disease. Our data compensate in part for the incomplete understanding of the metabolomics of the human PFC resulting from the difficulty of studying that organ in humans. Therefore, this research provides data and theoretical support for the discovery of diagnostic biomarkers and therapeutic targets for depression and lays a foundation for elucidating the pathophysiological mechanism of depression. However, there are still several limitations. First, we only tested the cortex, while other parts of the brain related to depression were not examined. Second, characterizing the underlying pathogenesis of depression will require further exploration.

## Data Availability Statement

The original contributions presented in the study are included in the article/Supplementary Materials, further inquiries can be directed to the corresponding author/s.

## Ethics Statement

The animal study was reviewed and approved by Animal Ethics Committee of the Institution of Research Animal Care of Central South University (approval ID: 2020SYDW0892). Written informed consent was obtained from the owners for the participation of their animals in this study.

## Author Contributions

CZ, RF, and TL contributed to the research design. ZY, WL, and JT performed the animal experiments. LD, TL, and EH acquired and analyzed the data. ZY participated in the experimental verification work. LD drafted and revised the manuscript. CZ and RF funded the study. All authors have read and approved the final manuscript.

## Funding

This work was supported by the National Natural Science Foundation of China (No. 81673951), Youth Foundation of National Natural Science Foundation of China (No. 81403259), and Natural Science Foundation of Hunan Province, China (No. 2017JJ2308).

## Conflict of Interest

The authors declare that the research was conducted in the absence of any commercial or financial relationships that could be construed as a potential conflict of interest.

## Publisher's Note

All claims expressed in this article are solely those of the authors and do not necessarily represent those of their affiliated organizations, or those of the publisher, the editors and the reviewers. Any product that may be evaluated in this article, or claim that may be made by its manufacturer, is not guaranteed or endorsed by the publisher.

## References

[1] Difrancesco S Penninx B Riese H Giltay EJ Lamers F. The role of depressive symptoms and symptom dimensions in actigraphy-assessed sleep, circadian rhythm, and physical activity. Psychol Med. (2021) 12:1–7. 10.1017/S0033291720004870 33431104 PMC9647516

[2] Kobau R Cui W Zack MM. Adults with an epilepsy history fare significantly worse on positive mental and physical health than adults with other common chronic conditions-Estimates from the 2010 National Health Interview Survey and Patient Reported Outcome Measurement System (PROMIS) Global Health Scale. Epilepsy Behav. (2017) 72:182–4. 10.1016/j.yebeh.2017.04.047 28606686 PMC6528480

[3] Malhi GS Mann JJ. Depression. Lancet. (2018) 392:2299–312. 10.1016/S0140-6736(18)31948-2 30396512

[4] Clark SM Pocivavsek A Nicholson JD Notarangelo FM Langenberg P McMahon RP. Reduced kynurenine pathway metabolism and cytokine expression in the prefrontal cortex of depressed individuals. J Psychiatry Neurosci. (2016) 41:386–94. 10.1503/jpn.150226 27070351 PMC5082509

[5] Koenigs M Grafman J. The functional neuroanatomy of depression: distinct roles for ventromedial and dorsolateral prefrontal cortex. Behav Brain Res. (2009) 201:239–43. 10.1016/j.bbr.2009.03.004 19428640 PMC2680780

[6] Komuro Y Oyama K Hu L Sakatani K. Relationship between cognitive dysfunction and systemic metabolic disorders in elderly: dementia might be a systematic disease. Adv Exp Med Biol. (2020) 1232:91–7. 10.1007/978-3-030-34461-0_13 31893399

[7] Veeraiah P Noronha JM Maitra S Bagga P Khandelwal N Chakravarty S. Dysfunctional glutamatergic and gamma-aminobutyric acidergic activities in prefrontal cortex of mice in social defeat model of depression. Biol Psychiatry. (2014) 76:231–8. 10.1016/j.biopsych.2013.09.024 24239130

[8] Duman RS Deyama S Fogaca MV. Role of BDNF in the pathophysiology and treatment of depression: activity-dependent effects distinguish rapid-acting antidepressants. Eur J Neurosci. (2021) 53:126–39. 10.1111/ejn.14630 31811669 PMC7274898

[9] Lu Q Mouri A Yang Y Kunisawa K Teshigawara T Hirakawa M. Chronic unpredictable mild stress-induced behavioral changes are coupled with dopaminergic hyperfunction and serotonergic hypofunction in mouse models of depression. Behav Brain Res. (2019) 372:112053. 10.1016/j.bbr.2019.112053 31288060

[10] Lu X Ce Q Jin L Zheng J Sun M Tang X. Deoiled sunflower seeds ameliorate depression by promoting the production of monoamine neurotransmitters and inhibiting oxidative stress. Food Funct. (2021) 12:573–86. 10.1039/D0FO01978J 33367360

[11] Belleau EL Treadway MT Pizzagalli DA. The impact of stress and major depressive disorder on hippocampal and medial prefrontal cortex morphology. Biol Psychiatry. (2019) 85:443–53. 10.1016/j.biopsych.2018.09.031 30470559 PMC6380948

[12] Huang X Li W You B Tang W Gan T Feng C. Serum metabonomic study on the antidepressant-like effects of ellagic acid in a chronic unpredictable mild stress-induced mouse model. J Agric Food Chem. (2020) 68:9546–56. 10.1021/acs.jafc.0c02895 32786855

[13] Chen G Yang D Yang Y Li J Cheng K Tang G. Amino acid metabolic dysfunction revealed in the prefrontal cortex of a rat model of depression. Behav Brain Res. (2015) 278:286–92. 10.1016/j.bbr.2014.05.027 24861712

[14] Eck H P Droge W. Influence of the extracellular glutamate concentration on the intracellular cyst(e)ine concentration in macrophages and on the capacity to release cysteine. Biol Chem Hoppe Seyler. (1989) 370:109–13. 10.1515/bchm3.1989.370.1.109 2641207

[15] Zhong P Zhang J Cui X. Abnormal metabolites related to bone marrow failure in aplastic anemia patients. Genet Mol Res. (2015) 14:13709–18. 10.4238/2015.October.28.33 26535686

[16] Rinschen MM Ivanisevic J Giera M Siuzdak G. Identification of bioactive metabolites using activity metabolomics. Nat Rev Mol Cell Biol. (2019) 20:353–67. 10.1038/s41580-019-0108-4 30814649 PMC6613555

[17] Michely JA Maurer HH. A multi-analyte approach to help in assessing the severity of acute poisonings - development and validation of a fast LC-MS/MS quantification approach for 45 drugs and their relevant metabolites with one-point calibration. Drug Test Anal. (2018) 10:164–76. 10.1002/dta.2257 28777878

[18] Wang LS Zhang MD Tao X Zhou YF Liu XM Pan RL. LC-MS/MS-based quantification of tryptophan metabolites and neurotransmitters in the serum and brain of mice. J Chromatogr B Analyt Technol Biomed Life Sci. (2019) 1112:24–32. 10.1016/j.jchromb.2019.02.021 30836315

[19] Han XM Qin YJ Zhu Y Zhang XL Wang NX Rang Y. Development of an underivatized LC-MS/MS method for quantitation of 14 neurotransmitters in rat hippocampus, plasma and urine: application to CUMS induced depression rats. J Pharm Biomed Anal. (2019) 174:683–95. 10.1016/j.jpba.2019.06.043 31288191

[20] Liu XJ Li ZY Li ZF Gao XX Zhou YZ Sun HF. Urinary metabonomic study using a CUMS rat model of depression. Magn Reson Chem. (2012) 50:187–92. 10.1002/mrc.2865 22367791

[21] Dong H Gao Z Rong H Jin M Zhang X. beta-asarone reverses chronic unpredictable mild stress-induced depression-like behavior and promotes hippocampal neurogenesis in rats. Molecules. (2014) 19:5634–49. 10.3390/molecules19055634 24786848 PMC6270931

[22] Wang S Xu X Ju X Wang S Li J Yan P. Melatonin ameliorated CUMS-induced depression-like behavior via restoring endoplasmic reticulum stress in rat hippocampus. Neuroreport. (2021) 32:8–15. 10.1097/WNR.0000000000001554 33165196

[23] Daodee S Monthakantirat O Ruengwinitwong K Gatenakorn K Maneenet J Khamphukdee C. Effects of the ethanol extract of dipterocarpus alatus leaf on the unpredictable chronic mild stress-induced depression in ICR mice and its possible mechanism of action. Molecules. (2019) 24:3396. 10.3390/molecules24183396 31540539 PMC6767234

[24] Li J Hou L Wang C Jia X Qin X Wu C. Short term intrarectal administration of sodium propionate induces antidepressant-like effects in rats exposed to chronic unpredictable mild stress. Front Psychiatry. (2018) 9:454. 10.3389/fpsyt.2018.00454 30319461 PMC6170646

[25] Daniel DG Daniel NG Daniel DT Flynn LC Allen MH. The effect of propofol on a forced swim test in mice at 24 hours. Curr Ther Res Clin Exp. (2020) 92:100590. 10.1016/j.curtheres.2020.100590 32714472 PMC7378852

[26] Hu E Ding R Li T Li P Feng D Hu W. Temporal metabolomic alteration in rat brains of experimental intracerebral hemorrhage. Brain Res Bull. (2021) 170:234–45. 10.1016/j.brainresbull.2021.02.021 33631271

[27] Zheng F Zhou YT Li PF Hu E Li T Tang T. Metabolomics analysis of hippocampus and cortex in a rat model of traumatic brain injury in the subacute phase. Front Neurosci. (2020) 14:876. 10.3389/fnins.2020.00876 33013291 PMC7499474

[28] Antoniuk S Bijata M Ponimaskin E Wlodarczyk J. Chronic unpredictable mild stress for modeling depression in rodents: meta-analysis of model reliability. Neurosci Biobehav Rev. (2019) 99:101–16. 10.1016/j.neubiorev.2018.12.002 30529362

[29] Sasibhushana RB Shankaranarayana Rao BS Srikumar BN. Repeated finasteride administration induces depression-like behavior in adult male rats. Behav Brain Res. (2019) 365:185–9. 10.1016/j.bbr.2019.03.006 30836157

[30] Zheng P Zeng B Zhou C Liu M Fang Z Xu X. Gut microbiome remodeling induces depressive-like behaviors through a pathway mediated by the host's metabolism. Mol Psychiatry. (2016) 21:786–96. 10.1038/mp.2016.44 27067014

[31] Winter G Hart RA Charlesworth RPG Sharpley CF. Gut microbiome and depression: what we know and what we need to know. Rev Neurosci. (2018) 29:629–43. 10.1515/revneuro-2017-0072 29397391

[32] Zhong S Zhang S Fan X Wu Q Yan L Dong J. A single-cell RNA-seq survey of the developmental landscape of the human prefrontal cortex. Nature. (2018) 555:524–8. 10.1038/nature25980 29539641

[33] Zhang F Chen H Zhang R Liu Y Kong N Guo Y. 5-Fluorouracil induced dysregulation of the microbiome-gut-brain axis manifesting as depressive like behaviors in rats. Biochim Biophys Acta Mol Basis Dis. (2020) 1866:165884. 10.1016/j.bbadis.2020.165884 32574836

[34] Zhang F Jia Z Gao P Kong H Li X Lu X. Metabonomics study of urine and plasma in depression and excess fatigue rats by ultra fast liquid chromatography coupled with ion trap-time of flight mass spectrometry. Mol Biosyst. (2010) 6:852–61. 10.1039/b914751a 20567771

[35] Przygodzki T Kazmierczak P Sikora J Watala C. 1-methylnicotinamide effects on the selected markers of endothelial function, inflammation and haemostasis in diabetic rats. Eur J Pharmacol. (2010) 640:157–62. 10.1016/j.ejphar.2010.05.014 20519141

[36] Kannt A Rajagopal S Kadnur SV Suresh J Bhamidipati RK Swaminathan S. A small molecule inhibitor of Nicotinamide N-methyltransferase for the treatment of metabolic disorders. Sci Rep. (2018) 8:3660. 10.1038/s41598-018-22081-7 29483571 PMC5826917

[37] Chlopicki S Swies J Mogielnicki A Buczko W Bartus M Lomnicka M. 1-Methylnicotinamide (MNA), a primary metabolite of nicotinamide, exerts anti-thrombotic activity mediated by a cyclooxygenase-2/prostacyclin pathway. Br J Pharmacol. (2007) 152:230–9. 10.1038/sj.bjp.0707383 17641676 PMC1978255

[38] Kuchmerovska T Shymanskyy I Chlopicki S Klimenko A. 1-methylnicotinamide (MNA) in prevention of diabetes-associated brain disorders. Neurochem Int. (2010) 56:221–8. 10.1016/j.neuint.2009.10.004 19837120

[39] Brites D Fernandes A. Neuroinflammation and depression: microglia activation, extracellular microvesicles and microRNA dysregulation. Front Cell Neurosci. (2015) 9:476. 10.3389/fncel.2015.00476 26733805 PMC4681811

[40] Zhao J Zhang Y Liu Y Tang WQ Ji CH Gu JH. Antidepressant-like effects of 1-methylnicotinamide in a chronic unpredictable mild stress model of depression. Neurosci Lett. (2021) 742:135535. 10.1016/j.neulet.2020.135535 33248165

[41] Mu RH Tan YZ Fu LL Nazmul Islam M Hu M Hong H. 1-Methylnicotinamide attenuates lipopolysaccharide-induced cognitive deficits via targeting neuroinflammation and neuronal apoptosis. Int Immunopharmacol. (2019) 77:105918. 10.1016/j.intimp.2019.105918 31639616

[42] Yanez M Jhanji M Murphy K Gower RM Sajish M Jabbarzadeh E. Nicotinamide augments the anti-inflammatory properties of resveratrol through PARP1 activation. Sci Rep. (2019) 9:10219. 10.1038/s41598-019-46678-8 31308445 PMC6629694

[43] Zinellu A Sotgia S Pisanu E Scanu B Sanna M Deiana L. Quantification of histidine, 1-methylhistidine and 3-methylhistidine in plasma and urine by capillary electrophoresis UV-detection. J Sep Sci. (2010) 33:3781–5. 10.1002/jssc.201000392 20886517

[44] Hu Z Fan S Liu M Zhong J Cao D Zheng P. Objective diagnosis of post-stroke depression using NMR-based plasma metabonomics. Neuropsychiatr Dis Treat. (2019) 15:867–81. 10.2147/NDT.S192307 31118636 PMC6498396

[45] Nakahara T Tsugawa S Noda Y Ueno F Honda S Kinjo M. Glutamatergic and GABAergic metabolite levels in schizophrenia-spectrum disorders: a meta-analysis of (1)H-magnetic resonance spectroscopy studies. Mol Psychiatry. (2021). 10.1038/s41380-021-01297-6. [Epub ahead of print]. 34584230

[46] Weckmann K Deery MJ Howard JA Feret R Asara JM Dethloff F. Ketamine's effects on the glutamatergic and GABAergic systems: a proteomics and metabolomics study in mice. Mol Neuropsychiatry. (2019) 5:42–51. 10.1159/000493425 31019917 PMC6465744

[47] Sabec MH Wonnacott S Warburton EC Bashir ZI. Nicotinic acetylcholine receptors control encoding and retrieval of associative recognition memory through plasticity in the medial prefrontal cortex. Cell Rep. (2018) 22:3409–15. 10.1016/j.celrep.2018.03.016 29590611 PMC5896173

[48] Wallace TL Bertrand D. Importance of the nicotinic acetylcholine receptor system in the prefrontal cortex. Biochem Pharmacol. (2013) 85:1713–20. 10.1016/j.bcp.2013.04.001 23628449

[49] Picciotto MR Higley MJ Mineur YS. Acetylcholine as a neuromodulator: cholinergic signaling shapes nervous system function and behavior. Neuron. (2012) 76:116–29. 10.1016/j.neuron.2012.08.036 23040810 PMC3466476

[50] Li H Li Y Zhang X Ren G Wang L Li J. The Combination of Aquilaria sinensis (Lour.) Gilg and Aucklandia costus Falc. volatile oils exerts antidepressant effects in a CUMS-induced rat model by regulating the HPA Axis and levels of neurotransmitters. Front Pharmacol. (2020) 11:614413. 10.3389/fphar.2020.614413 33716727 PMC7943885

[51] Higley MJ Picciotto MR. Neuromodulation by acetylcholine: examples from schizophrenia and depression. Curr Opin Neurobiol. (2014) 29:88–95. 10.1016/j.conb.2014.06.004 24983212 PMC4268065

[52] Risch SC Cohen RM Janowsky DS Kalin NH Murphy DL. Mood and behavioral effects of physostigmine on humans are accompanied by elevations in plasma beta-endorphin and cortisol. Science. (1980) 209:1545–6. 10.1126/science.7433977 7433977

[53] Schulz JM Reynolds JN. Pause and rebound: sensory control of cholinergic signaling in the striatum. Trends Neurosci. (2013) 36:41–50. 10.1016/j.tins.2012.09.006 23073210

[54] Mark GP Rada PV Shors TJ. Inescapable stress enhances extracellular acetylcholine in the rat hippocampus and prefrontal cortex but not the nucleus accumbens or amygdala. Neuroscience. (1996) 74:767–74. 10.1016/0306-4522(96)00211-4 8884772

[55] Whalen PJ Kapp BS Pascoe JP. Neuronal activity within the nucleus basalis and conditioned neocortical electroencephalographic activation. J Neurosci. (1994) 14:1623–33. 10.1523/JNEUROSCI.14-03-01623.1994 8126559 PMC6577570

[56] Simon GM Cravatt BF. Anandamide biosynthesis catalyzed by the phosphodiesterase GDE1 and detection of glycerophospho-N-acyl ethanolamine precursors in mouse brain. J Biol Chem. (2008) 283:9341–9. 10.1074/jbc.M707807200 18227059 PMC2431036

[57] Topuz RD Cetinkaya MZ Erumit D Duvan Aydemir K Gunduz O Karadag CH. The role of endocannabinoid system and TRPV1 receptors in the antidepressant and anxiolytic effects of dipyrone in chronic unpredictable mild stress in mice. Eur J Pharmacol. (2021) 908:174315. 10.1016/j.ejphar.2021.174315 34270988

[58] Fernandez-Ruiz J. The endocannabinoid system as a target for the treatment of motor dysfunction. Br J Pharmacol. (2009) 156:1029–40. 10.1111/j.1476-5381.2008.00088.x 19220290 PMC2697699

[59] Rodriguez WA Horne CA Mondragon AN Phelps DD. Comparable dose-response functions for the effects of glucose and fructose on memory. Behav Neural Biol. (1994) 61:162–9. 10.1016/S0163-1047(05)80070-6 8204081

[60] Sapolsky RM. Glucocorticoid toxicity in the hippocampus: reversal by supplementation with brain fuels. J Neurosci. (1986) 6:2240–4. 10.1523/JNEUROSCI.06-08-02240.1986 3746406 PMC6568753

[61] Liu YY Zhou XY Yang LN Wang HY Zhang YQ Pu JC. Social defeat stress causes depression-like behavior with metabolite changes in the prefrontal cortex of rats. PLoS One. (2017) 12:e0176725. 10.1371/journal.pone.0176725 28453574 PMC5409051

[62] Belujon P Grace AA. Dopamine system dysregulation in major depressive disorders. Int J Neuropsychopharmacol. (2017) 20:1036–46. 10.1093/ijnp/pyx056 29106542 PMC5716179

[63] Drevets WC Zarate CA Jr Furey ML. Antidepressant effects of the muscarinic cholinergic receptor antagonist scopolamine: a review. Biol Psychiatry. (2013) 73:1156–63. 10.1016/j.biopsych.2012.09.031 23200525 PMC4131859

[64] Janowsky DS el-Yousef MK Davis JM. Acetylcholine and depression. Psychosom Med. (1974) 36:248–57. 10.1097/00006842-197405000-00008 4829619

