# Supplementary figures and images for: Metabolomics Analysis of the Prefrontal Cortex in a Rat Chronic Unpredictable Mild Stress Model of Depression
Source: Front Psychiatry. 2022 Mar 15;13:815211. doi: 10.3389/fpsyt.2022.815211 (PMC8965009; doi:10.3389/fpsyt.2022.815211)

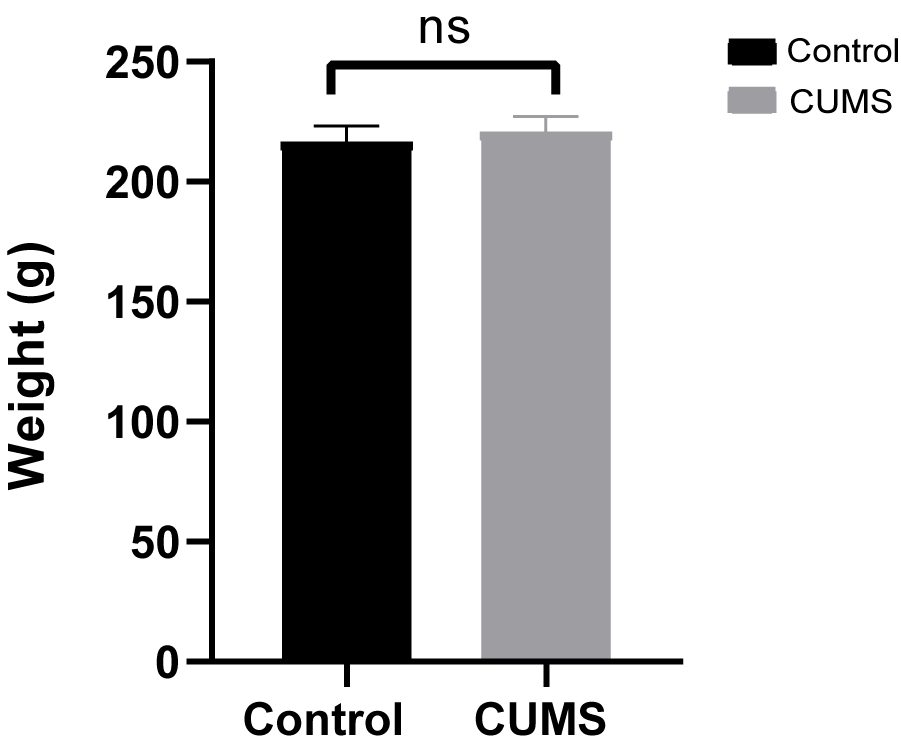

Supplement: Supplementary file 1 [file Data_Sheet_1.ZIP › Figure S/Fig S1.tif]

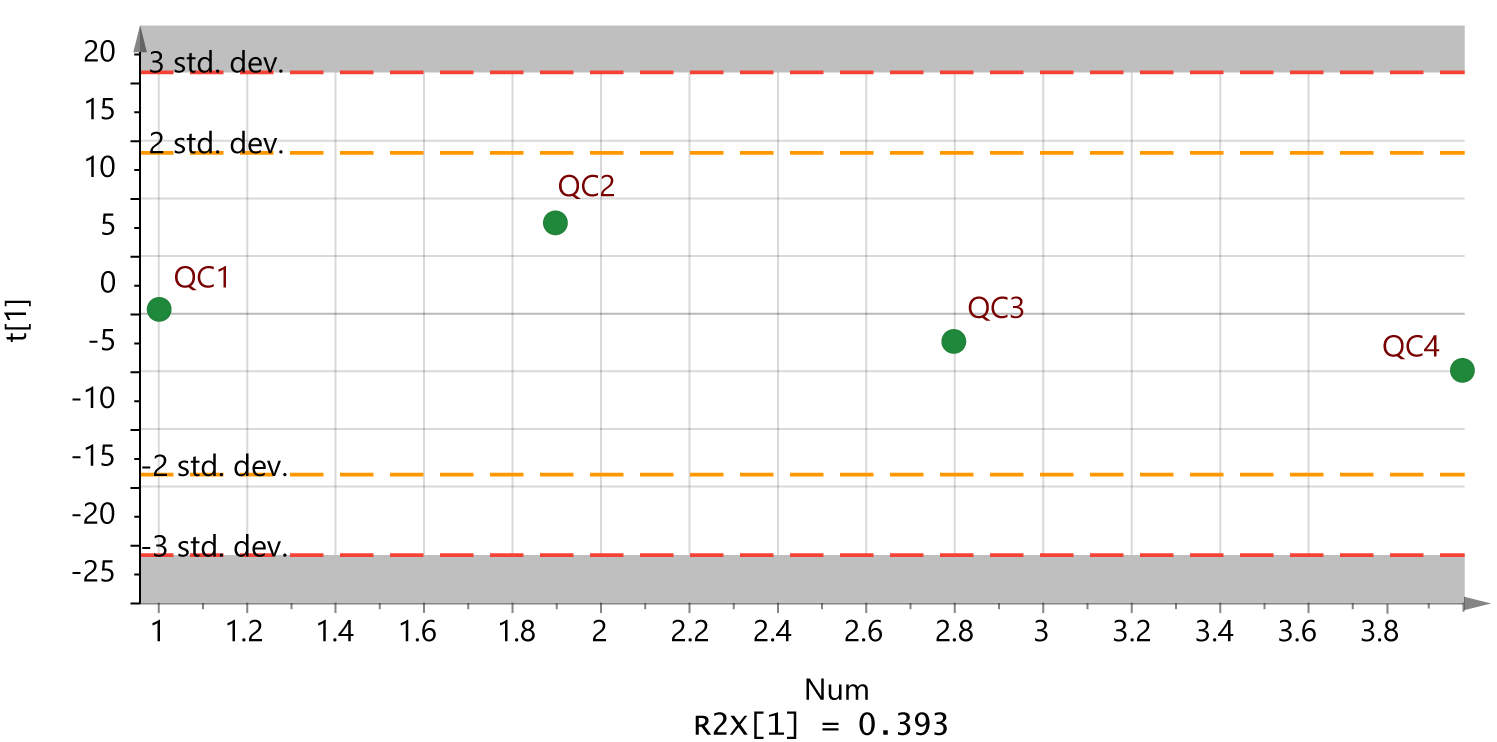

Supplement: Supplementary file 1 [file Data_Sheet_1.ZIP › Figure S/Fig S2.tif]

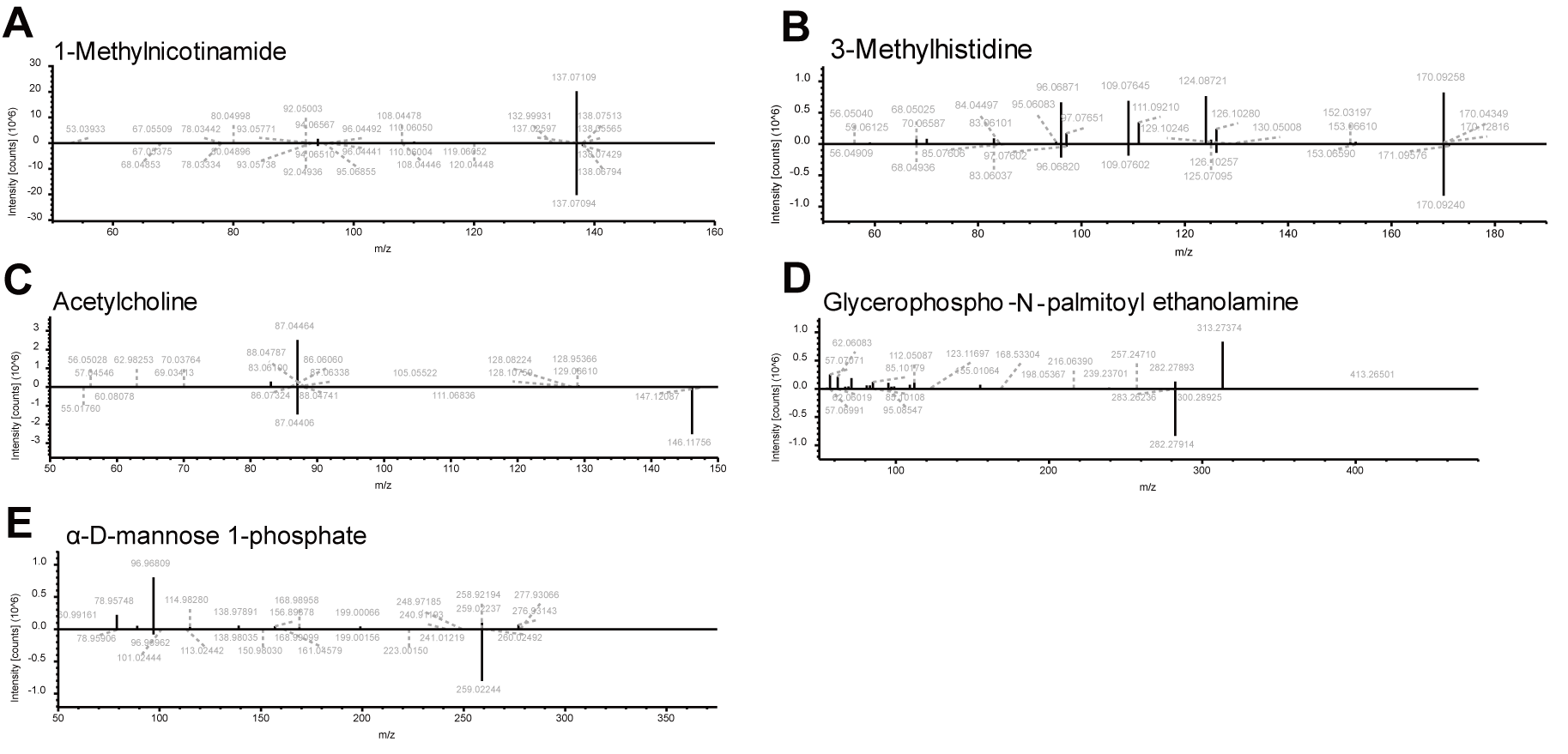

Supplement: Supplementary file 1 [file Data_Sheet_1.ZIP › Figure S/Fig S3.tif]

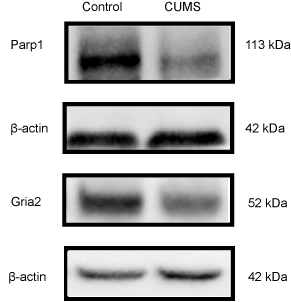

Supplement: Supplementary file 1 [file Data_Sheet_1.ZIP › Figure S/Fig S4.tif]
