# Supplementary material for: Metabolomics Analysis of the Prefrontal Cortex in a Rat Chronic Unpredictable Mild Stress Model of Depression
Source: Front Psychiatry. 2022 Mar 15;13:815211. doi: 10.3389/fpsyt.2022.815211 (PMC8965009; doi:10.3389/fpsyt.2022.815211)
Supplement: Supplementary file 1 [file Data_Sheet_1.ZIP › Figure S/Supplementary table 1.doc]

| **SUPPLEMENTARY TABLE 1**. Significant enriched metabolic pathways. | | | | |
| --- | --- | --- | --- | --- |
| **Pathway Name** | ***P* value** | **Impact** | **Hits/ total** | **Hits** |
| Histidine metabolism | 0.05 | 0.00 | 1/16 | 3-Methylhistidine |
| Nicotinate and nicotinamide metabolism | 0.04 | 0.14 | 1/15 | 1-Methylnicotinamide |
| Glycerophospholipid metabolism | 0.11 | 0.00 | 1/36 | Acetylcholine |
| Fructose and mannose metabolism | 0.05 | 0.12 | 1/18 | α-D-Mannose 1-phosphate |
| Amino sugar and nucleotide sugar metabolism | 0.117 | 0.06 | 1/37 | α-D-Mannose 1-phosphate |
