# Supplementary material for: Metabolomics Analysis of the Prefrontal Cortex in a Rat Chronic Unpredictable Mild Stress Model of Depression
Source: Front Psychiatry. 2022 Mar 15;13:815211. doi: 10.3389/fpsyt.2022.815211 (PMC8965009; doi:10.3389/fpsyt.2022.815211)
Supplement: Supplementary file 1 [file Data_Sheet_1.ZIP › Figure S/Supplementary table 2.doc]

| **SUPPLEMENTARY TABLE 2**. Significantly enriched pathways of metabolites related proteins. | | | | |
| --- | --- | --- | --- | --- |
| **KEGG ID** | **Pathway Name** | **Term *P*** | **Hits/ total** | **Hits** |
| 04080 | Neuroactive ligand-receptor interaction | 3.85975E-15 | 4.40 | [ADRB2, CHRM2, CHRM3, CHRNA2, CHRNA7, CNR1, GCG, GRIA1, GRIA2, GRIA3, GRIA4, GRIK2, GRIK3, LPAR1, LPAR3] |
| 04725 | Cholinergic synapse | 2.06432E-05 | 4.42 | [ACHE, CHAT, CHRM2, CHRM3, CHRNA7] |
| 04713 | Circadian entrainment | 0.000202228 | 4.12 | [GRIA1, GRIA2, GRIA3, GRIA4] |
| 04723 | Retrograde endocannabinoid signaling | 4.49236E-06 | 4.05 | [CNR1, FAAH, GRIA1, GRIA2, GRIA3, GRIA4] |
| 04724 | Glutamatergic synapse | 9.74602E-07 | 5.26 | [GRIA1, GRIA2, GRIA3, GRIA4, GRIK2, GRIK3] |
| 04728 | Dopaminergic synapse | 9.85454E-08 | 5.30 | [GRIA1, GRIA2, GRIA3, GRIA4, GSK3B, SLC6A3, TH] |
| 04730 | Long-term depression | 0.000793427 | 5.00 | [GRIA1, GRIA2, GRIA3] |
| 05030 | Cocaine addiction | 0.000436862 | 6.12 | [GRIA2, SLC6A3, TH] |
| 05031 | Amphetamine addiction | 4.7992E-08 | 8.70 | [GRIA1, GRIA2, GRIA3, GRIA4, SLC6A3, TH] |
| 05033 | Nicotine addiction | 1.12554E-07 | 12.50 | [CHRNA7, GRIA1, GRIA2, GRIA3, GRIA4] |
